# Cognitive Correlates of a Novel Simulated Online Money Management Credit Card Task

**DOI:** 10.1093/geroni/igaf122.106

**Published:** 2025-12-31

**Authors:** Preeti Sunderaraman, Madison Bouchard-Liporto, Silvia Chapman, Sandra Rizer, Masanao Yajima, Stephanie Cosentino

**Affiliations:** Memory and Aging Program, Butler Hospital; Brown University, Boston, Massachusetts, United States; Boston University - Chobanian & Avedisian School of Medicine, Boston, Massachusetts, United States; Columbia University Irving Medical Center, New York, New York, United States; Columbia University Irving Medical Center, New York, New York, United States; Boston University Department of Mathematics and Statistics, Boston, Massachusetts, United States; Columbia University Irving Medical Center, New York, New York, United States

## Abstract

About 65% of older adults experience credit card fraud accounting for loss of $246 million. As older adults are also at risk for cognitive decline, it becomes necessary to develop novel tools to assess online money management (OMM) credit card skills. This study examined the convergent and divergent validity of a novel, simulated OMM credit card task designed to replicate real-world scenarios. Data were collected from 190 participants (M age (yrs)=67.84; SD=4.26, M education (yrs)=16.65, SD=2.26, 69.5% female, 89.5% White). The OMM task comprises of: i) Online Navigation (ON); logging into a simulated credit card account to download a statement, ii) Simple Literacy (SL); clicking on answers in response to questions about the statement, and iii) Statement Monitoring (SM); identifying erroneous transactions. Metrics for each component included number of clicks and time taken to complete each task; additionally, number of correct (accuracy) was present for SL and SM. Bivariate correlations examined the association between OMM components, NIH toolbox tests and the FunStatsTM battery components of online banking and ATM. OMM metrics related to ON and SL were most strongly associated with online banking, while SM was most strongly associated with auditory memory. Compared to other OMM metrics, SL accuracy showed more associations with cognitive abilities. This study found evidence for convergent and divergent validity of the OMM task. These findings are promising and indicate that the OMM task is useful to assess various aspects of OMM credit card skills and those at risk for financial exploitation.

